# Pancreatic rendezvous technique for treating a disconnected pancreatic duct syndrome in a patient with ansa pancreatica

**DOI:** 10.1055/a-2433-1247

**Published:** 2024-10-25

**Authors:** Jean Grimaldi, Antoine Guilloux, Xavier Dray, Marine Camus Duboc, Romain Leenhardt, Mathieu Pioche, Ulriikka Chaput

**Affiliations:** 1 Endoscopy and Gastroenterology Unit, Edouard Herriot Hospital, Hospices Civils de Lyon, Lyon, France; 2 Endoscopy Unit, Sorbonne University, Saint Antoine Hospital, Paris, France

We present the case of a 36-year-old patient with disconnected pancreatic duct syndrome following acute necrotizing alcoholic pancreatitis. As the patient experienced recurrent pancreatic collections, endoscopic retrograde cholangiopancreatography was performed to bridge the pancreatic disconnection and prevent further collection recurrence.

The initial step involved catheterizing the main pancreatic duct (MPD) via the major papilla ( Video 1 ). Opacification of the MPD revealed the presence of an ansa pancreatica ( Fig. 1 ), along with contrast leakage from the body portion of the MPD, confirming the disconnection. Owing to the significant angulation associated with the ansa pancreatica, the guidewire repeatedly ascended into the Santorini duct but failed to enter the body portion of the MPD ( Fig. 2 ). A pancreatic stent was placed in the MPD, but migrated into the duodenum during the procedure.

Pancreatic rendezvous technique in a patient with ansa pancreatica.Video 1

**Fig. 1:**
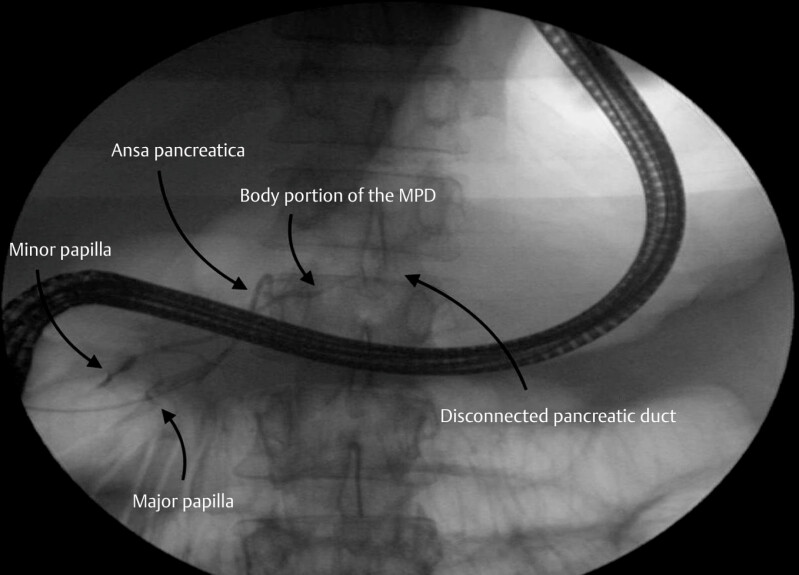
The first part of the procedure was to catheterize the main pancreatic duct (MPD).

**Fig. 2:**
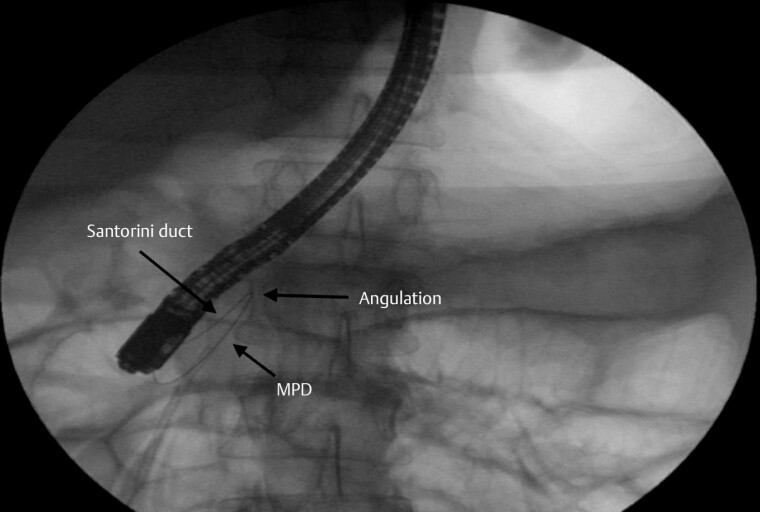
Owing to the significant angulation associated with the ansa pancreatica, the guidewire repeatedly ascended into the Santorini duct but failed to enter the body portion of the main pancreatic duct (MPD).

As catheterization of the minor papilla was unsuccessful, we decided to perform a pancreatic rendezvous technique by catheterizing the Santorini duct from the major papilla. The guidewire was advanced through the minor papilla ( Fig. 3 ). As the attempt to catheterize the minor papilla along with the previously inserted guidewire failed, it was then decided to capture the guidewire with a snare, and retrieve it through the working channel of the duodenoscope. The guidewire was then introduced into a sphincterotome, which was thus able to catheterize the minor papilla. This approach allowed the body portion of the MPD to be catheterized ( Fig. 4 ), enabling the placement of a plastic stent to reach the disconnected area. The procedure was completed without complications.

**Fig. 3:**
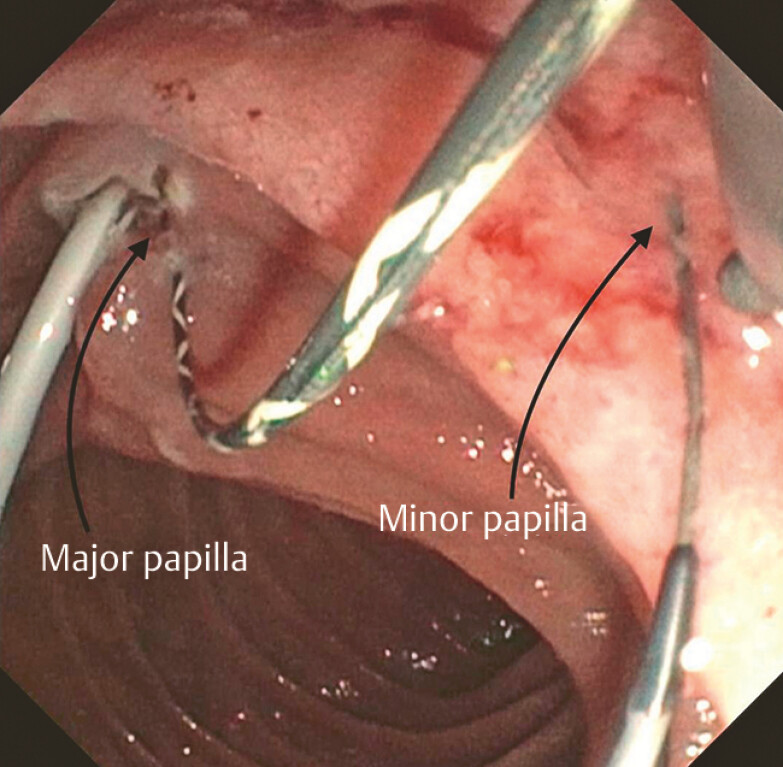
The guidewire was advanced through the minor papilla.

**Fig. 4:**
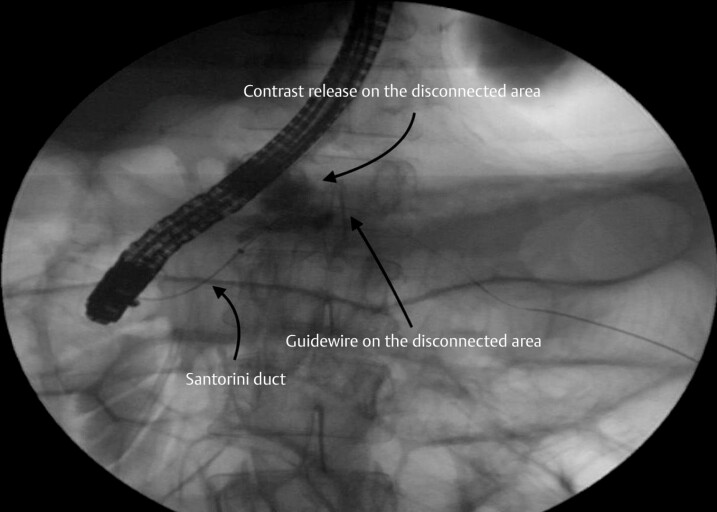
This approach allowed the body portion of the main pancreatic duct to be catheterized, enabling the placement of a plastic stent to reach the disconnected area.

Although the pancreatic rendezvous technique has been described previously 1 , it is now most commonly performed via the endoscopic ultrasound approach 2 3 . The technique described herein avoids the morbidity associated with endoscopic ultrasound-guided pancreatic puncture and appears to be preferable in cases involving altered anatomy, such as ansa pancreatica.

Endoscopy_UCTN_Code_TTT_1AR_2AG

## Notes

Endoscopy E-Videos https://eref.thieme.de/e-videos
*E-Videos* is an open access online section of the journal *Endoscopy* , reporting on interesting cases and new techniques in gastroenterological endoscopy. All papers include a high-quality video and are published with a Creative Commons CC-BY license. Endoscopy E-Videos qualify for HINARI discounts and waivers and eligibility is automatically checked during the submission process. We grant 100% waivers to articles whose corresponding authors are based in Group A countries and 50% waivers to those who are based in Group B countries as classified by Research4Life (see: https://www.research4life.org/access/eligibility/ ). This section has its own submission website at https://mc.manuscriptcentral.com/e-videos .
